# Decompression of a Mandibular Dentigerous Cyst Using a Dual‐Purpose Removable Appliance in a Pediatric Patient: A Case Report

**DOI:** 10.1155/crid/8724204

**Published:** 2026-04-12

**Authors:** Buse Nur Gok, Saffet Ugur Dogan, Askin Dilara Kaynak, Mehmet Ali Altay, Ozge Erken Gungor

**Affiliations:** ^1^ Department of Pediatric Dentistry, Akdeniz University Faculty of Dentistry, Antalya, Türkiye, akdeniz.edu.tr; ^2^ Department of Oral and Maxillofacial Surgery, Akdeniz University Faculty of Dentistry, Antalya, Türkiye, akdeniz.edu.tr

**Keywords:** decompression treatment, dentigerous cyst, mixed dentition, removable appliance, space maintainer

## Abstract

Dentigerous cysts (DCs) are among the most common odontogenic lesions in mixed dentition. Management strategies vary from radical enucleation—often necessitating the removal of associated tooth germs—to more conservative decompression techniques. In pediatric patients, decompression is preferred as it preserves developing permanent teeth and supports normal eruption. In January 2023, an 8.5‐year‐old male presented to the Department of Pediatric Dentistry, Akdeniz University, with swelling in the right mandibular region. Clinical and radiographic examination revealed a unilocular radiolucent lesion associated with primary teeth #84 and #85. These teeth were extracted under local anesthesia, and the clinical, radiographic, and histopathological findings were considered most consistent with a DC showing secondary inflammation. During the same session, a removable acrylic appliance with drainage channels was fabricated from a conventional impression. The patient irrigated the lesion twice daily with saline while wearing the appliance. At 1 month, radiographs showed a reduction in lesion size. After 9 months, tooth #44 erupted into normal position, and the appliance was modified to function as a space maintainer until the eruption of the premolars. Decompression is frequently preferred for treating DC in children; however, silicone drains may reduce comfort and hygiene. In this case, a removable acrylic appliance provided decompression and was subsequently modified to function as a space maintainer. Such removable acrylic appliances may offer practical advantages in selected pediatric patients by facilitating hygiene, supporting eruption, and allowing subsequent space maintenance.

## 1. Introduction

In the mixed dentition phase, dentigerous cysts (DCs) represent a significant portion of developmental odontogenic pathologies [1]. They are benign intraosseous lesions, often asymptomatic, and typically located in the mandibular region [2]. DCs are generally associated with unerupted permanent teeth or developing tooth buds. Pathogenesis involves fluid accumulation between the reduced enamel epithelium and the crown of a developing permanent tooth. Radiographic presentation typically consists of a distinctly demarcated, single‐chambered radiolucent area, often surrounded by a sclerotic margin [3].

Management options include enucleation, which often requires the extraction of the associated impacted teeth, and decompression or marsupialization [4]. In pediatric patients, decompression is generally preferred because it preserves developing permanent teeth and facilitates their eruption [5]. This technique maintains a small opening in the cyst wall for continuous drainage, reducing intracystic pressure. Acrylic stents or silicone tubes fixed with sutures are commonly used to keep the cyst–oral cavity communication open [6]. However, silicone drains can accumulate plaque, increase infection risk, delay healing, and compromise comfort.

This case report describes a modified appliance‐based decompression approach using a removable acrylic appliance with drainage channels in a pediatric patient with a mandibular cystic lesion most consistent with a DC. The appliance was designed to facilitate cleaning and improve patient comfort; it was later modified to function as a space maintainer. This case report was prepared in accordance with the CARE guidelines, and the completed CARE Checklist is provided as Supporting Information 1.

## 2. Case Description

**Figure 1:**
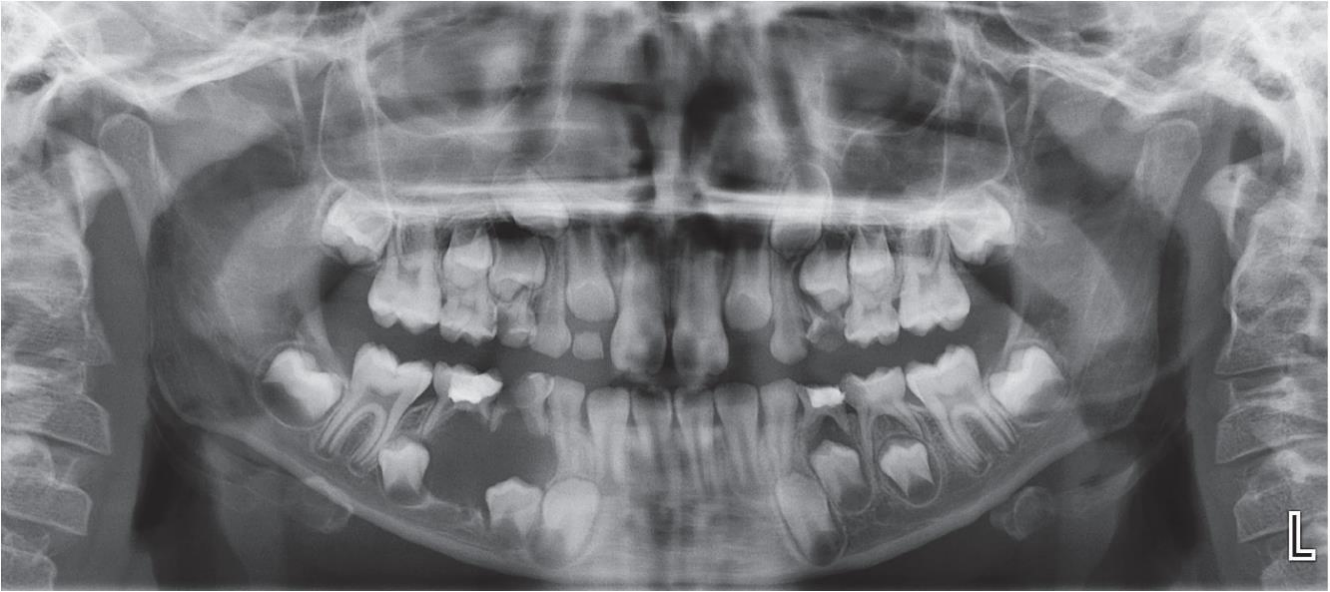
Pretreatment panoramic radiograph showing apical displacement of teeth #44 and #45 toward the inferior border of the mandible.

**Figure 2:**
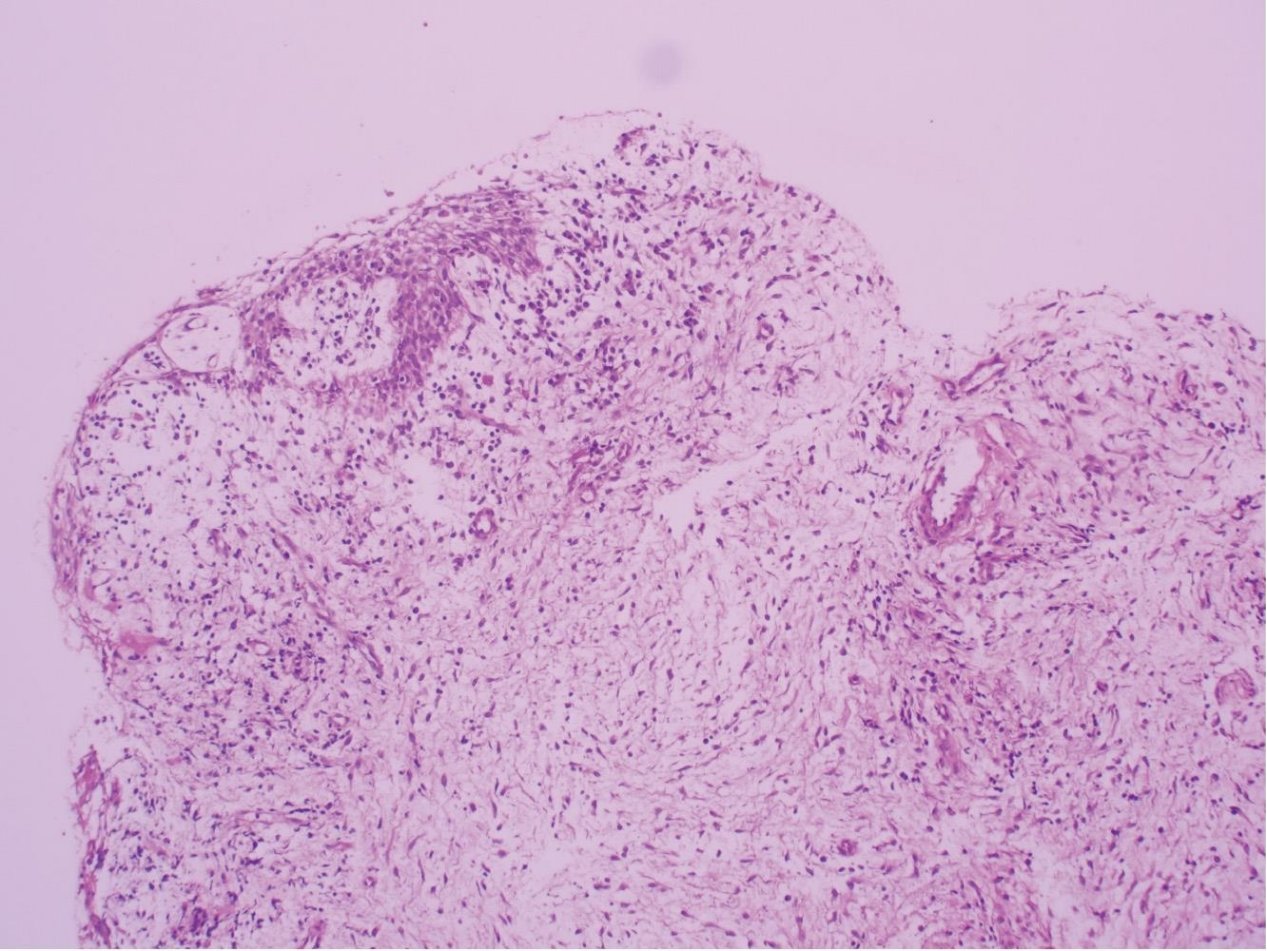
Photomicrograph showing a cystic lesion lined with stratified squamous epithelium exhibiting focal erosion and dense chronic inflammatory infiltration (H&E, ×100).

An 8.5‐year‐old male patient presented to the Department of Pediatric Dentistry, Akdeniz University, in January 2023 with a painless swelling in the right mandibular region. Radiographic examination revealed a well‐defined unilocular radiolucency extending from the distal aspect of tooth #83 to the mesial aspect of tooth #46, located apically to primary teeth #85 and #84, and involving the entire crown of tooth #44 and part of the crown of tooth #45 (Figure 1). Intraoral examination confirmed buccal swelling adjacent to primary teeth #84 and #85. Medical history revealed that tooth #85 had undergone pulpotomy in another clinic in 2022. Approximately 6 months later, the patient experienced spontaneous pain, for which antibiotic therapy was prescribed; however, no further dental treatment was provided. Panoramic radiographs showed deep caries, furcation radiolucency, and distal root resorption in tooth #84 (Figure 1). Given the chronic course of symptoms and the lesion’s association with tooth #85, pulp vitality testing was performed on teeth #84 and #85, both of which yielded negative responses to electric pulp and cold testing. Based on the clinical and radiographic findings, the lesion was considered suggestive of a DC. However, distinction from a radicular cyst associated with the nonvital primary teeth remained challenging, even after histopathological examination. Microscopic analysis revealed a thin, nonkeratinized stratified squamous epithelial lining and a fibrous capsule with diffuse chronic inflammatory cell infiltration. Together with the radiographic relationship of the lesion to the crown of tooth #44, these findings were considered most consistent with a DC showing secondary inflammation (Figure 2).

Prior to surgery, written and verbal informed consent was obtained from the patient’s parents. Primary teeth #85 and #84, suspected to be associated with the cyst, were extracted under inferior alveolar nerve block supplemented with a buccal infiltration (ULTRAVER D‐S, Haver, Turkey). Following extraction, the lesion was accessed intraorally, and an incisional biopsy was obtained for histopathological analysis. To avoid injury to the developing permanent teeth, the cystic cavity was not curetted; instead, cystic fluid was aspirated and the cavity irrigated with sterile saline. The specimen was placed in a transport medium and submitted to the pathology department. Sterile iodoform gauze was placed in the cystic cavity to prevent leakage of the impression material. A conventional impression was taken using C‐type silicone (Kulzer Soft Putty, Kulzer, Germany). The patient was prescribed amoxicillin (500 mg twice daily for 7 days) and paracetamol (500 mg, as needed for pain control) as part of the postoperative care following extraction, biopsy, and initiation of decompression.

**Figure 3:**
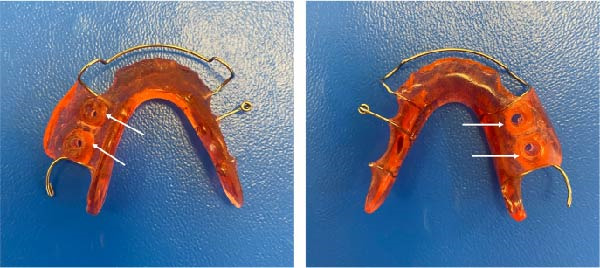
Removable acrylic appliance used for decompression. White arrows indicate the acrylic drainage channels extending toward the extraction site/cystic cavity and maintaining communication for saline irrigation.

Postoperative instructions were explained to the parents. A plaster model was fabricated from the silicone impression, and a removable acrylic appliance with drainage channels extending 3.5 mm into the lesion site was constructed (Figure 3). Histopathological examination supported the clinic and radiographic impression of a DC showing secondary inflammation. At the follow‐up visit 1 day postextraction, the appliance was delivered, and the parents were instructed to ensure its use for at least 22 h/day and to irrigate the lesion twice daily with saline through the drainage channels.

The patient was reviewed at 1‐week intervals. At each visit, appliance fit, oral hygiene, soft tissue healing, and patient comfort were assessed. At the 1‐month follow‐up, clinical and radiographic evaluation demonstrated bone regeneration within the previous cystic area, improved eruption trajectories of the first and second premolars, and progression of the tooth buds toward occlusion (Figure 4a).

Figure 4(a) Panoramic radiograph at 1‐month follow‐up. (b) Panoramic radiograph at 2‐month follow‐up.

**Figure:**
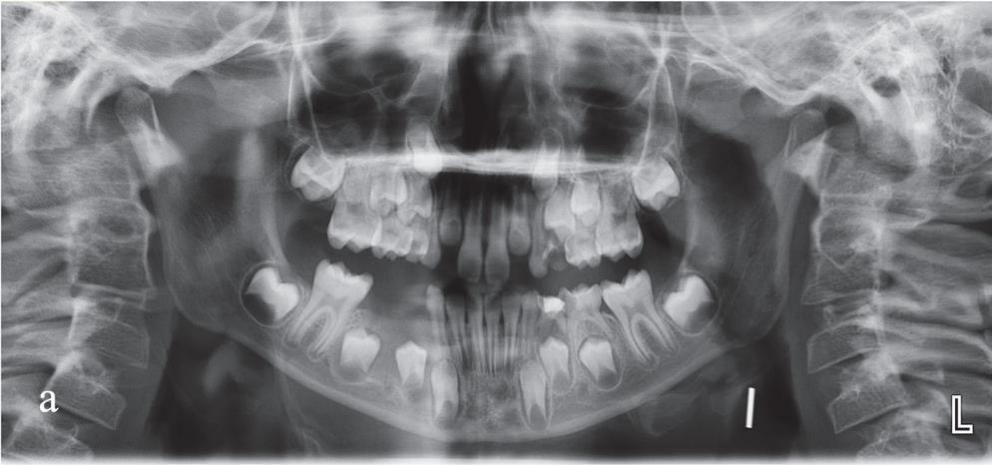
(a)

**Figure:**
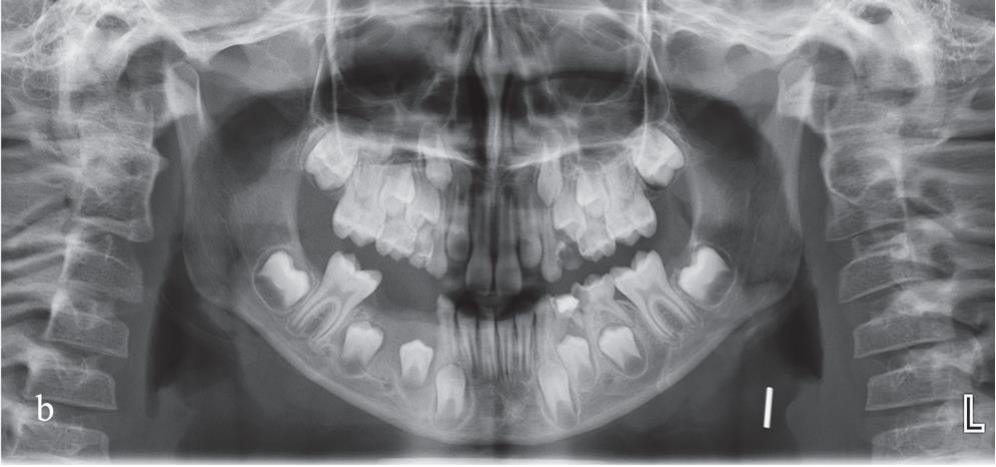
(b)

To objectively monitor radiographic change over time, linear measurements were performed on digital panoramic radiographs. Lesion measurements were performed by a single investigator on baseline and follow‐up digital panoramic radiographs obtained using the same device, the same patient positioning protocol, and the same software settings. The initial lesion area was estimated as ~361.18 mm^2^ (22.73 mm × 15.89 mm). At the 1‐month follow‐up, the estimated area had decreased to 40.29 mm^2^ (9.48 mm × 4.25 mm), corresponding to an approximate 88.8% reduction on 2D panoramic assessment.

**Figure 6:**
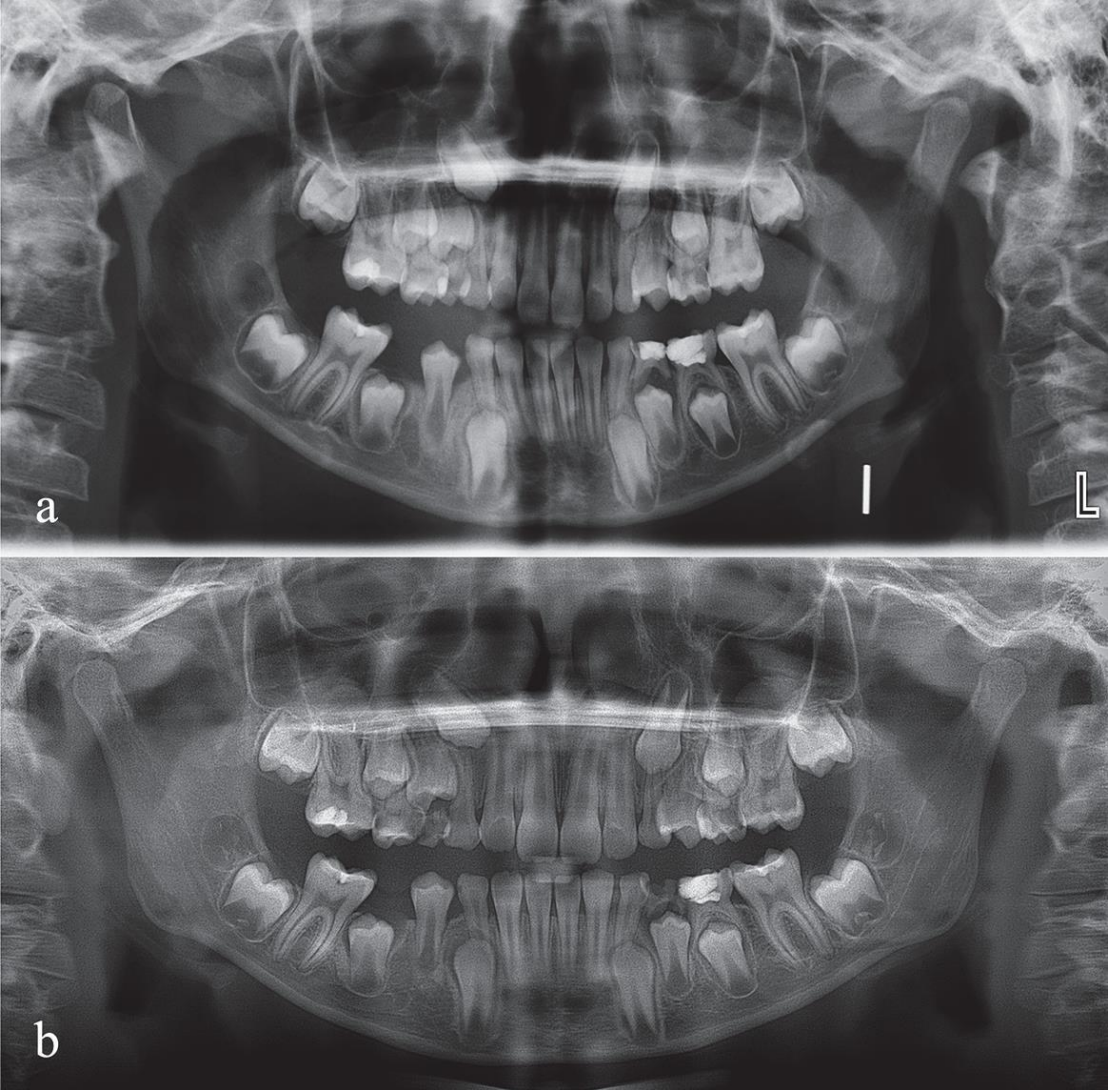
(a) Radiographic resolution observed at 12‐month follow‐up. (b) Complete healing and final radiograph at 18‐month follow‐up.

Gingival healing at the extraction site and intraoral appliance positioning at this stage are shown in Figure 5. At the end of the first month, decompression was deemed complete, and the appliance was modified by removing the acrylic drainage channels with an aerator under water cooling, allowing it to function as a space maintainer. At the 2‐month follow‐up, panoramic radiography confirmed preservation of the space between teeth #46 and #83 (Figure 4b). At the 10‐month follow‐up, further modification of the appliance was performed in the region of tooth #44 to accommodate its eruption while maintaining space for tooth #45. By the 12‐month follow‐up, tooth #44 had fully erupted, and the eruption path of tooth #45 showed marked improvement (Figure 6a). Eighteen months after the initial decompression, complete radiographic resolution of the cystic lesion was observed, coinciding with the eruption of tooth #44. The appliance was discontinued, and treatment was concluded (Figure 6b).

Figure 5(a) Intraoral appearance of the appliance at 1‐month follow‐up. (b) Soft tissue healing at the extraction site after the decompression phase.

**Figure:**
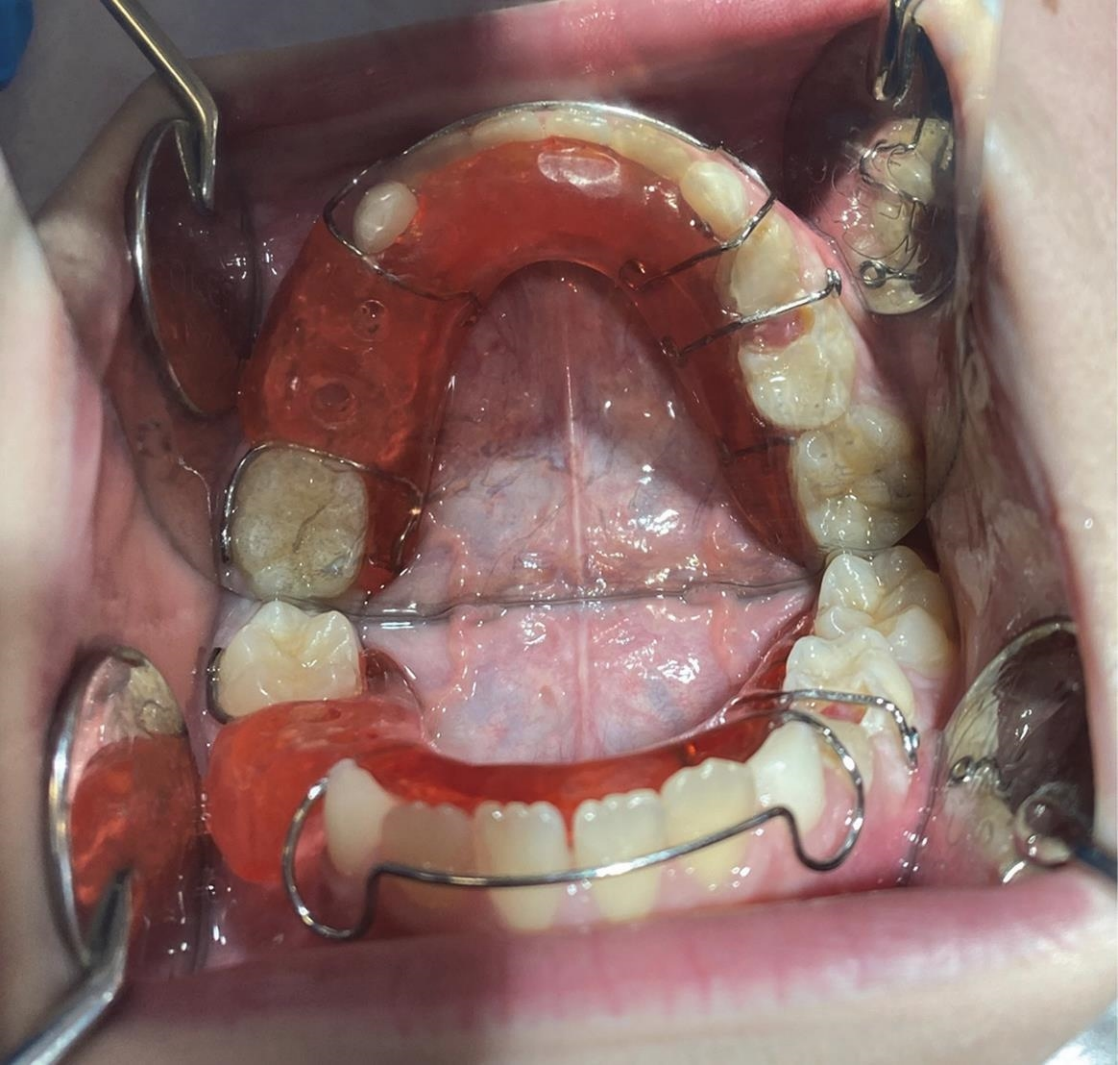
(a)

**Figure:**
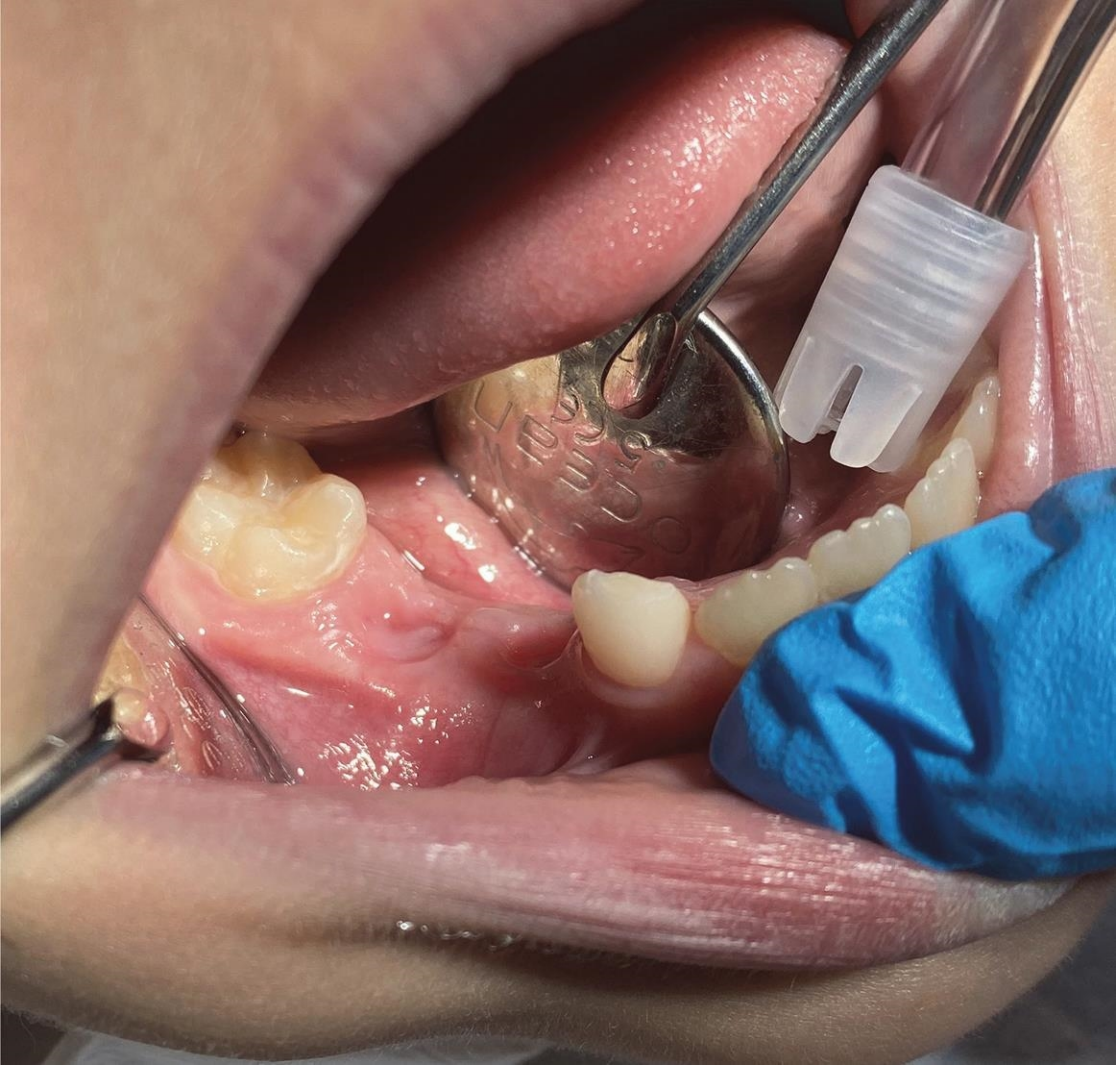
(b)

## 3. Discussion

The differential diagnosis between a radicular cyst associated with a primary tooth and a DC involving its successor can be challenging, particularly when nonvital primary teeth are present. In the present case, the lesion’s attachment near the cemento‐enamel junction of tooth #44 and the displacement of the developing premolars favored a DC; however, this distinction could not be considered definitive because clinical, radiographic, and histopathological findings may overlap in secondarily inflamed DCs and radicular cysts. For this reason, the lesion is more appropriately described as being most consistent with a DC showing secondary inflammation. The term “inflammatory dentigerous cyst” is used here only in the descriptive sense reported in the pediatric literature [7–11] and not as a distinct pathological entity in the current WHO classification.

Decompression is a well‐established conservative approach for managing DC in pediatric patients [12]. It offers particular advantages in the mixed dentition by preserving developing permanent teeth, facilitating physiological eruption, and reducing the risk of damage to adjacent anatomical structures. Conventional decompression methods, which typically involve silicone drains, acrylic stents, or plastic tubes fixed with sutures, may compromise patient comfort, increase plaque accumulation, and delay healing [13, 14]. Furthermore, suture loosening or breakage can necessitate re‐suturing, prolonging treatment duration and increasing infection risk.

In the present case, these limitations were addressed through the use of a removable acrylic appliance with drainage channels. This design maintained continuous communication between the cystic cavity and the oral environment without the need for sutures and may have facilitated hygiene and patient comfort by allowing extraoral cleaning by the caregivers during follow‐up. The appliance was fabricated with the intention of subsequent modification into a space maintainer once decompression was complete, thereby preserving arch length and supporting the eruption of teeth #44 and #45.

The concept of combining decompression with space maintenance has been described previously, and the present approach should therefore be interpreted as a practical modification rather than a completely novel technique. Chouchene et al. [15] reported a DC showing secondary inflammation in the mixed dentition managed with the extraction of the associated primary teeth and the immediate fabrication of an acrylic obturator, which maintained cyst patency and acted as a space maintainer. This resulted in complete healing and spontaneous premolar eruption within 10 months. Similarly, Berberi et al. [2] documented a mandibular DC in a 9‐year‐old managed with a fixed silicone drain later replaced by a space maintainer, achieving bone regeneration at 6 months and complete healing at 5 years. In this context, the present approach may be regarded as a practical modification in which decompression and subsequent space maintenance were managed with the same removable appliance, which could be progressively adjusted during eruption.

Evidence supporting the clinical usefulness of conservative decompression in pediatric odontogenic cystic lesions is substantial. Uloopi et al. [16] advocated marsupialization for large radicular cysts associated with nonvital primary teeth, involving the creation of a surgical window in the buccal cortical plate and maintenance of cavity patency with a drain or acrylic obturator. This approach preserved permanent teeth, reduced cyst size, prevented food impaction, and promoted bone regeneration, with subsequent use of space maintainers. Allon et al. [6] in a retrospective study of 26 children (32 lesions; 62.5% DC), reported a mean treatment duration of 7.45 ± 2.6 months, with an average lesion size reduction of 82% ± 16% and complete resolution in many cases without secondary surgery. Their protocol involved fenestration with iodoform gauze or polyethylene stents and regular irrigation. In the present case, lesion reduction and eruption were achieved without a separate second‐stage surgical procedure, suggesting that appliance‐based decompression may be a clinically useful conservative option in selected cases.

Factors influencing spontaneous eruption following DC treatment have also been investigated. Nahajowski et al. [17] identified younger age (~10 years), root development ≤ 1/2, cusp depth, tooth angulation, cyst area, and available eruption space as positive predictors, with ~62% of premolars erupting spontaneously after conservative treatment, despite low evidence quality. Pei et al. [18] evaluated 25 radicular cysts in 23 children and found an average lesion size reduction rate of 0.77 ± 0.44 cm^2^/month, with larger lesions (>3.5 cm^2^) showing faster reduction. All successor teeth erupted, although 46% demonstrated root development anomalies. In the present report, the patient was 8.5 years old, with root development ≤½, preserved space, and favorable angulation, resulting in the timely eruption of #44 within 9–12 months, consistent with these predictive factors.

Long‐term outcomes and appliance design have also been addressed in the literature. Sevekar et al. [19] demonstrated that a modified Hawley appliance could maintain decompression, promote bone regeneration, preserve space, and reduce malocclusion risk, while improving comfort and reducing clinic visits. Similarly, a retrospective analysis [5] involving 34 pediatric mandibular DC cases reported a mean decompression duration of 5.97 months (range 3–9) using a customized Hawley plate with a stainless‐steel drainage tube, without major complications. In the present report, decompression and space maintenance were achieved without the use of metal tubing, which may represent a practical alternative in selected cooperative pediatric patients. Ahmed and Kaushal [20] also described two large radicular cysts managed conservatively with marsupialization and decompression, reporting preservation of permanent teeth and favorable long‐term follow‐up outcomes. Although the pathology in the present report was more consistent with a DC than a radicular cyst, the conservative treatment principles are comparable.

## 4. Limitations

This report is limited to a single clinical case, and the findings should therefore be interpreted cautiously. The success of the removable appliance also depends heavily on patient and caregiver compliance, as it requires consistent daily wear of at least 22 h and regular irrigation of the cystic cavity.

In addition, the quantitative radiographic assessment was based on 2D panoramic images obtained at baseline and follow‐up and measured by a single investigator using the same device, the same patient positioning protocol, and the same software settings. However, panoramic imaging remains subject to magnification, positioning error, and image distortion, and repeated measurements or intraobserver reliability assessment were not performed. Accordingly, the reported reduction should be interpreted as an approximate comparative estimate rather than a true volumetric assessment of healing. Larger clinical series are needed to better define the indications, limitations, and reproducibility of such dual‐purpose appliances in pediatric patients.

## 5. Conclusion

This case suggests that conservative decompression with a removable acrylic appliance may be a feasible treatment option in selected pediatric patients with cystic lesions most consistent with a DC. In the present patient, the approach was associated with marked radiographic reduction and favorable eruption of the involved premolar. It also allowed decompression without sutured drain fixation and later served as a space maintainer. Although interpretation is limited by the single‐case design and 2D panoramic follow‐up, the technique appears to be a practical conservative option in appropriately selected cases.

## Author Contributions

Buse Nur Gok was involved in conceptualization, methodology, formal analysis, investigation, visualization, and writing the original draft. Saffet Ugur Dogan contributed to methodology, resources, and investigation. Askin Dilara Kaynak participated in the investigation, data curation, and resources. Mehmet Ali Altay was responsible for validation and supervision. Ozge Erken Gungor contributed to conceptualization, methodology, project administration, supervision, and writing – review and editing.

## Funding

The authors confirm that the study was conducted independently and without any financial support from public, commercial, or not‐for‐profit funding agencies.

## Disclosure

All authors have read and approved the final manuscript. The authors would like to note that this case was previously presented as a poster at the FDI World Dental Congress in 2024. The abstract of the poster was published in the *International Dental Journal*, Volume 74, Supplement, 2024, page 354 (DOI: 10.1016/j.identj.2024.07.448). However, this case has not been published as a full‐length article previously.

## Ethics Statement

This report describes the routine clinical management of a patient who presented for dental treatment. As the procedures performed were part of a standard treatment protocol and did not constitute a preplanned research study, formal institutional ethical committee approval was not required at the time of initiation. However, the study was conducted in accordance with the principles of the Declaration of Helsinki.

## Consent

Written and verbal informed consent was obtained from the patient’s parents and legal guardians for the treatment, and specific consent was secured for the use of clinical photographs, radiographic images, and histopathological specimens for academic publication.

## Conflicts of Interest

The authors declare no conflicts of interest.

## Supporting Information

Additional supporting information can be found online in the Supporting Information section.

## Supporting information


**Supporting Information** 1. CARE Checklist completed for the present case report, in accordance with the CARE guidelines.

## Data Availability

The data supporting the findings of this case report are available within the article. Additional anonymized patient information is available from the corresponding author upon reasonable request.
