# Supplementary material for: Decompression of a Mandibular Dentigerous Cyst Using a Dual‐Purpose Removable Appliance in a Pediatric Patient: A Case Report
Source: Case Rep Dent. 2026 Apr 12;2026:8724204. doi: 10.1155/crid/8724204 (PMC13071175; doi:10.1155/crid/8724204)
Supplement: Supplementary file 1 — Supporting Information 1. CARE Checklist completed for the present case report, in accordance with the CARE guidelines. [file CRID-2026-8724204-s001.pdf]

CARE case report guidelines

CARE Checklist of information to include when writing a case report

(cc) BY-NC-ND

Topic	Item	Checklist item description		Reported on Line
Title	1	The diagnosis or intervention of primary focus followed by the words "case report"	✓	Page 1, Line 1-2
Key Words	2	2 to 5 key words that identify diagnoses or interventions in this case report, including "case report"	✓	Page 2, Line 56-57
Abstract (no references)	3a	Introduction: What is unique about this case and what does it add to the scientific literature?	✓	Page 2, Line 50-52
	3b	Main symptoms and/or important clinical findings	✓	Page 2, Line 41-42
	3c	The main diagnoses, therapeutic interventions, and outcomes	✓	Page 2, Line 43-48
	3d	Conclusion—What is the main "take-away" lesson(s) from this case?	✓	Page 2, Line 49-54
Introduction	4	One or two paragraphs summarizing why this case is unique (**may include references**)	✓	Page 2, Line 59-76
Patient Information	5a	De-identified patient specific information.	✓	Page 3, Line 79-80
	5b	Primary concerns and symptoms of the patient.	✓	Page 3, Line 80-84
	5c	Medical, family, and psycho-social history including relevant genetic information	✓	Page 3, Line 85-89
	5d	Relevant past interventions with outcomes	✓	Page 3, Line 85-89
Clinical Findings	6	Describe significant physical examination (PE) and important clinical findings.	✓	Page 3, Line 80-93
Timeline	7	Historical and current information from this episode of care organized as a timeline	✓	Page 4, Line 115-126
Diagnostic Assessment	8a	Diagnostic testing (such as PE, laboratory testing, imaging, surveys).	✓	Page 4 Line 107-108
	8b	Diagnostic challenges (such as access to testing, financial, or cultural)		Not applicable
	8c	Diagnosis (including other diagnoses considered)		Not applicable
	8d	Prognosis (such as staging in oncology) where applicable	✓	Discussion Section
Therapeutic Intervention	9a	Types of therapeutic intervention (such as pharmacologic, surgical, preventive, self-care)	✓	Page 4, Line 116-119
	9b	Administration of therapeutic intervention (such as dosage, strength, duration)	✓	Page 3-4, Line 94-110
	9c	Changes in therapeutic intervention (with rationale)	✓	Page 4, Line 116-119
Follow-up and Outcomes	10a	Clinician and patient-assessed outcomes (if available)	✓	Page 4, Line 115-126
	10b	Important follow-up diagnostic and other test results	✓	Page 4, Line 115-126
	10c	Intervention adherence and tolerability (How was this assessed?)	✓	Page 4, Line 108-126
	10d	Adverse and unanticipated events		Not applicable
Discussion	11a	A scientific discussion of the strengths AND limitations associated with this case report	✓	Page 4-5-6
	11b	Discussion of the relevant medical literature **with references**.	✓	Page 4-5-6
	11c	The scientific rationale for any conclusions (including assessment of possible causes)	✓	Page 4-5-6
	11d	The primary "take-away" lessons of this case report (without references) in a one paragraph conclusion	✓	Page 4-5-6
Patient Perspective	12	The patient should share their perspective in one to two paragraphs on the treatment(s) they received		Not reported
Informed Consent	13	Did the patient give informed consent? Please provide if requested		Yes ☑ No ☐
